# New Horizons for MXenes in Biosensing Applications

**DOI:** 10.3390/bios12100820

**Published:** 2022-10-02

**Authors:** Decheng Lu, Huijuan Zhao, Xinying Zhang, Yingying Chen, Lingyan Feng

**Affiliations:** 1 Department of Materials Genome Institute, Shanghai University, Shanghai 200444, China; 2 Qing Wei Chang College, Shanghai University, Shanghai 200444, China; 3 Shanghai Engineering Research Center of Organ Repair, Shanghai 200444, China

**Keywords:** two-dimensional material, MXenes, biosensor, electrochemistry, optics

## Abstract

Over the last few decades, biosensors have made significant advances in detecting non-invasive biomarkers of disease-related body fluid substances with high sensitivity, high accuracy, low cost and ease in operation. Among various two-dimensional (2D) materials, MXenes have attracted widespread interest due to their unique surface properties, as well as mechanical, optical, electrical and biocompatible properties, and have been applied in various fields, particularly in the preparation of biosensors, which play a critical role. Here, we systematically introduce the application of MXenes in electrochemical, optical and other bioanalytical methods in recent years. Finally, we summarise and discuss problems in the field of biosensing and possible future directions of MXenes. We hope to provide an outlook on MXenes applications in biosensing and to stimulate broader interests and research in MXenes across different disciplines.

## 1. Introduction

The main two-dimensional (2D) material is a solid crystal consisting of a single or several atomic layers, a sheet thickness of 1–10 Å, and a lateral size ranging from 100 nm to several μm [1]. Two-dimensional materials with properties such as large specific surface area and unique electronics are focuses of research in many research fields [2]. Since 2004, Novoselov et al. performed exfoliation to obtain graphene nanostructures; since then, the two-dimensional material has attracted much attention [3]. In 2011, Gogotsi et al. prepared a two-dimensional Ti_3_C_2_ nanosheet named MXenes [4]. MXenes are typically a few μm laterally and 1 nm thick or less [5]. It shows superior physicochemical properties compared to other two-dimensional nanomaterials [6].

**Figure 1:**
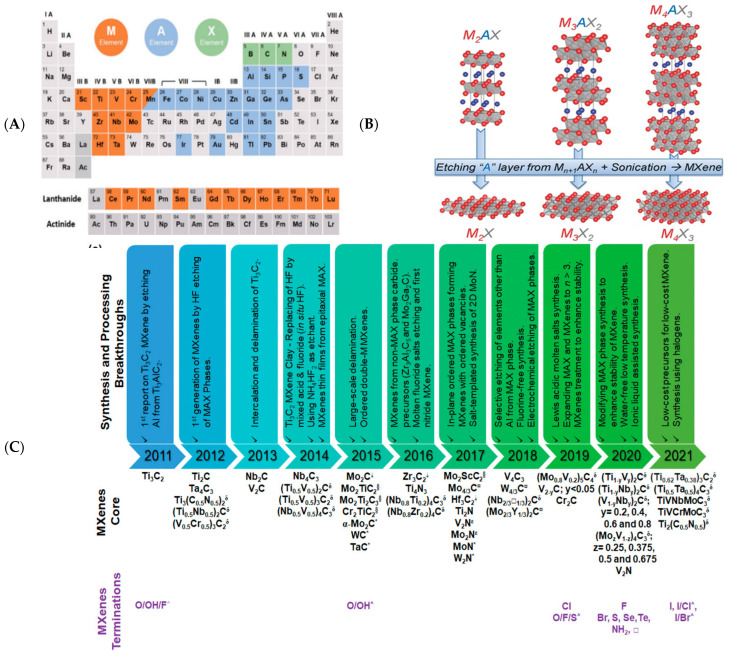
(**A**) Elements represented by M, A, and X in the MAX phase. (**B**) The structure of the MAX phase and its corresponding MXenes. Reprinted with permission from Ref. [9]. Copyright 2013, Wiley-VCH. (**C**) Chronological presentations of progress in the field of MXenes. List of the main synthesis and processing breakthroughs over the first 10 years of MXenes’ research and new MXenescore compositions discovered in that decade and progress in surface terminations control. ^δ^ Solid solution MXenes; ^□^ MXenesfrom non-MAX phase precursors; ^§^ out-of-plane ordered double transition metal MXene; ^¤^ MXenes from in-plane ordered double transition metal MAX phase analogues; * 2D carbides and nitrides produced by bottom-up approaches; ^ε^ nitride MXenes produced by the post-synthesis treatment of carbide MXene; ^□^ vacancy; ^^^ mixed terminations. Reprinted with permission from Ref. [12]. Copyright 2021, Wiley-VCH.

The precursor of MXenes is the MAX phase. MAX consists of M_n+1_X_n_ units and an alternately stacked “A” element single atomic plane, expressed as M_n+1_AX_n._ The unique crystal structure of the MAX phase combines the excellent properties of ceramics and metals [7]. Etching the “A” element of the MAX phase yields two-dimensional nanomaterial MXenes with a structural formula of M_n+1_X_n_T_x_ [8]. MXenes can be expressed as M_2_XF_2_, M_2_X(OH)_2_, M_2_XO_2_, etc. M is a transition metal; “A” is an element of Groups 13 and 14 of the periodic table; X is boron, carbon, or nitrogen; n includes integers from 1 to 3; T_x_ denotes surface groups [9] (Figure 1A,B). A list of the significant syntheses and processes in the field of MXenes research over the last decade, as well as the development of new MXenes core components and surface group control techniques, is illustrated in Figure 1C. Compared to the precursor MAX phase, derivative MXenes retain metallic and electrical conductivity benefits of MAX but also offer smaller lateral dimensions and thicknesses, as well as unique physical and chemical properties [10,11].

**Scheme 1:**
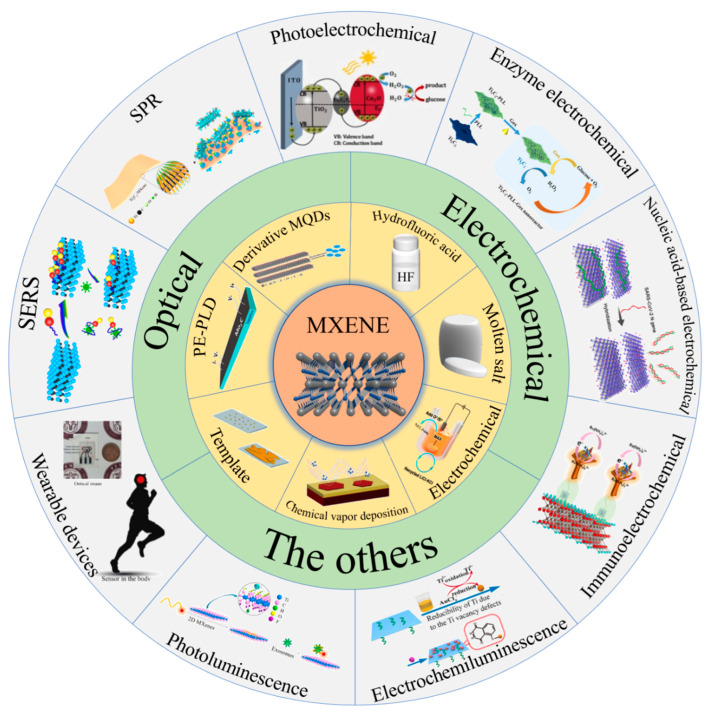
MXenes cover both top-down and bottom-up methods of synthesis. They play an irreplaceable position in enzyme-, nucleic-acid-, immune-based electrochemical biosensing; photoluminescence; electrochemiluminescence; photoelectric effect-based optical biosensors; and other biosensors such as wearable biosensors, surface plasmon resonance, and surface-enhanced Raman spectroscopy.

The central area of current advanced biosensing research studies is developing biosensors for detecting biological and chemical molecules that affect disease or are damaging to the human body. The most advanced biosensors can accurately and rapidly detect the target, predict the onset of the disease in time, and receive immediate medical attention [13]. Hence, high sensitivity and selectivity are significant for the design of biosensors. Due to its unique mechanical, hydrophilic, biocompatibility, and other excellent properties, MXenes are frequently used as a new biosensing platform. Electrochemical biosensors are essential for biological, environmental, and pharmaceutical fields. It offers high sensitivity, long-term reliability and high accuracy, rapidity, low cost, and easy miniaturisation [14]. In addition, electrochemical biosensors offer a further path for creating next-generation point-of-care testing devices [15]. With advancing nanotechnology with respect to MXene-based optical biosensors, unprecedented progress has been made in optical analysis. Optical analysis has advantages of high sensitivity, high selectivity, fast analysis, and good reproducibility. It has been widely used in biochemistry and biomedical and environmental analysis and has received increasing attention [16]. The synthesis of MXenes and their application in biosensing are reflected in Scheme 1. We will review and summarize published studies on biosensing since the development of MXenes, including those mainly classifying biosensors into electrochemical, optical biosensors and some derivative biosensors. In addition, we will also discuss the challenges of MXenes in preparing biosensors and future perspectives on applying MXenes in biosensing.

## 2. Synthesis and Structures of MXenes

### 2.1. Synthesis of MXenes

There are two methods for the synthesis of MXenes. The top-down method is the most commonly used, which can be used to exfoliate multilayer materials into a few-layer or single-layer MXenes sheet. The second method is a bottom-up approach, which focuses on the growth of Mxenes from atoms or molecules [17,18].

#### 2.1.1. Top-Down Method

**Figure 2:**
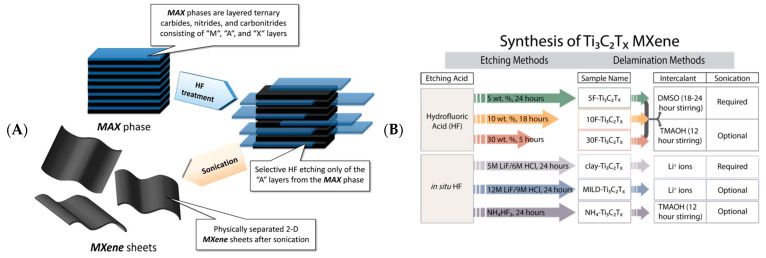
(**A**) Schematic diagram of the process of preparing MXenes by HF. Reprinted with permission from Ref. [21]. Copyright 2012, American Chemical Society. (**B**) A guide to Ti_3_C_2_ MXenes synthesis using HF. Reprinted with permission from Ref. [22]. Copyright 2017, American Chemical Society.

Selective etching disintegrates the strong covalent bonds between the MX and the A layers in the MAX phase. The primary method is etching with hydrofluoric acid (HF), molten salts, etc. In this process, oxygen (O), hydroxyl (OH), and fluorine (F) replace the M-A strong metal bond [17]. There are two main steps to gain 2D MXenes by HF: etching and exfoliation. Although the direct use of HF is straightforward and practical, it causes environmental pollution and damages to the human body [4]. In situ HF can be obtained by reacting a fluorinated salt with mild acid, which is less toxic to MXenes surfaces [19]. Researchers explored new synthetic methods (Figure 2). The typical chemical reaction equation for the synthesis of MXenes in the MAX phase is as follows [9].

(1) $$M_{n+1}{\mathrm{AX}}_{n}+3\mathrm{HF}\underset{}{\to }{\mathrm{AF}}_{3}+M_{n+1}X_{n}+\frac{3}{2}H_{2}$$

(2) $$M_{n+1}X_{n}+2H_{2}O\underset{}{\to }M_{n+1}X_{n}{\left(\mathrm{OH}\right)}_{2}+H_{2}$$

(3) $$M_{n+1}X_{n}+2\mathrm{HF}\underset{}{\to }M_{n+1}X_{n}F_{2}+H_{2}$$

MXenes must undergo an exfoliation process to obtain nanosheet structures: The surface groups of MXenes result in the layers being linked by hydrogen and Van der Waals forces [3]. Exfoliation enhances the interlayer spacing by weakening interactions between layers using various molecular and ionic processes [20].

**Figure 3:**
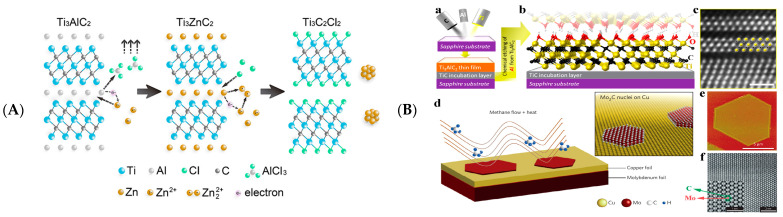
(**A**) Preparation mechanism of Ti_3_C_2_Cl_2_ etched by ZnCl_2_. Reprinted with permission from Ref. [30]. Copyright 2019, American Chemical Society. (**B**) Bottom-up approach to obtain MXenes. Atomic layer deposition method: steps to prepare Ti_3_AlC_2_ MAX films by sputtering Ti, Al and C on a sapphire substrate (**a**), schematic diagram of Ti_3_C_2_T_x_ (**b**) and STEM images (**c**). CVD method: schematic diagram of the Mo_2_C synthesis process (**d**), AFM images of hexagonal ultra-thin Mo_2_C crystals (**e**) and STEM images (**f**). Reprinted with permission from Ref. [27]. Copyright 2020, American Chemical Society.

The molten salt method uses fluorinated molten salts, Lewis salts [23]. The synthesis does not involve fluoride, reducing the risk of synthesis [8,24]. The mechanism of MXenes formation in molten salts is similar to that of conventional HF methods: ZnCl_2_ and CuCl_2_ high-temperature molten salts strip a more comprehensive range of MAX phase materials [8] (Figure 3A). In the molten salt of Lewis acids, Zn^2+^, Cu^2+^, and Cl^−^ are consistent with acting H^+^ and F^−^ in HF. Minimally intensive layer delamination (MILD) and electrochemical etching can also be used for MAX etching, producing high-quality, non-toxic MXenes [25,26].

#### 2.1.2. Bottom-Up Method

Bottom-up synthesis methods have been reported, such as chemical vapor deposition (CVD) [27], template [28], and plasma-enhanced pulsed laser deposition (PE-PLD) [29] (Figure 3B). MXenes produced by this method possess good crystalline quality and controllable structure and size [18].

Xu et al. used CVD to synthesize high-quality Mo_2_C crystals [27]. The synthesis of Mo_2_C MXene/graphene heterostructures and Mo_2_C MXene-graphene hybrid films by this method has been reported [29,31]. Compared to CVD, the template method has a relatively high yield of MXenes. Two-dimensional MXenes are mainly obtained by carbonizing or nitriding two-dimensional transition metal oxide (TMO) nanosheet templates. Xia et al. prepared hexagonal-structured 2D h-MoN nanosheets using precursor MoO_2_ nanosheets [28]. PE-PLD is a successful method for preparing large-area ultra-thin face-centered cubic (FCC) Mo_2_C MXene [29].

The stability of MXenes is an important property and limits its application to a certain extent. Researchers have tried to improve its stability. High concentrations of HF accelerate the degradation of MXenes and affect its structure, so relatively mild reaction conditions are necessary [32]. Organic solvents mitigate the oxidation of MXenes. Contact with water should be avoided as much as possible to prevent oxidation [33]. The oxidation of MXenes is quicker in liquid media than in solid media, and this degradation process is exacerbated by photocatalysis and thermocatalysis [34]. The storage of MXenes in Ar-sealed vials at 4 °C exhibits high stability at room temperatures [35].

### 2.2. Strustures of Mxenes

**Figure 4:**
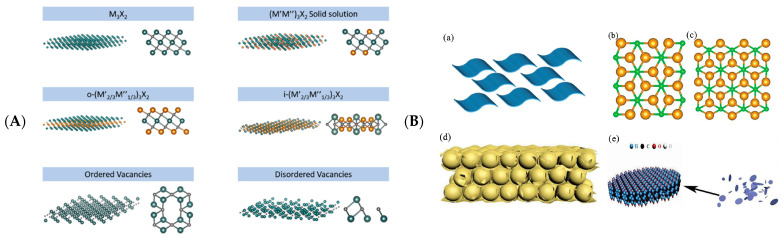
(**A**) Different types of MXenes structures. Reprinted with permission from Ref. [42]. Copyright 2019, Elsevier. (**B**) 2D, 1D, 3D, and 0D structures of MXenes. Adapted with permission from Ref. [18]. Copyright 2021, Wiley-VCH.

The crystal structure within a 2D material can affect its properties [18]. There are six types of MXenes structures (Figure 4A): (1) single transition metal MXenes (Ti_3_C_2_ and Nb_4_C_3_); (2) solid solution MXenes ((Ti, V)_3_C_2_ and (Cr, V)_3_C_2_); (3) sequential planar internal and external bimetal MXenes with one transition metal occupying the outer layer (Cr and Mo); the central metal is another metal (Nb and Ta) [36,37]; (4) ordered double-transition metals MXenes ((Cr_2_V) C_2_); (5) orrderly double vacancy MXenes (Mo_1.33_CTx) [38]; (6) random empty space MXenes (Nb_1.33_CTx) [39].

Computational simulation studies have been reported to identify novel stable MXenes structures, contributing to exploratory studies [40]. The properties and applications of these materials can be adapted by various parameters for composition, surface modification by heat treatment or chemical pathways, and structural adjustments [41]. MXenes have two—dimensional structures (a), one—dimensional structures (b) and (c), three—dimensional structures (d), and zero—dimensional structures (e) (Figure 4B).

## 3. MXenes in Biosensing

Several strategies involving MXenes in analytical nanoscience, biosensing, and other areas have been reported. MXenes exhibit hydrophilicity due to surface groups such as OH, O, and F. Its surface can interact with most biomolecules through hydrogen bonding, Van der Waals forces, electrostatic interactions, and ligand binding, rendering it an excellent carrier for biosensors applications [43,44,45]. Several different MXenes compositions have been proved to be biocompatible and non-cytotoxic [46,47].

We summarized the composition and analytical performance of some MXene-based electrochemical biosensors, optical biosensors, and other biosensors and attached them to the subsections. These cases demonstrate the broad applicability of MXenes in the fabrication of biosensors. Readers can easily extract MXene-based biosensing research and measurement data from these tables.

### 3.1. Electrochemical Biosensing

Due to their high electronic conductivity, MXenes can drive most electrochemical reactions, which is of great significance for the application in electrochemical biosensing [48]. The electrical properties of MXenes can be improved by changing elemental compositions and surface groups [18]. In particular, the external transition metal layer of MXenes plays a more critical role in the electronic properties than the internal layer [49]. The number and thickness of the layers of MXenes also affect electrical properties [3,50].

**Figure 5:**
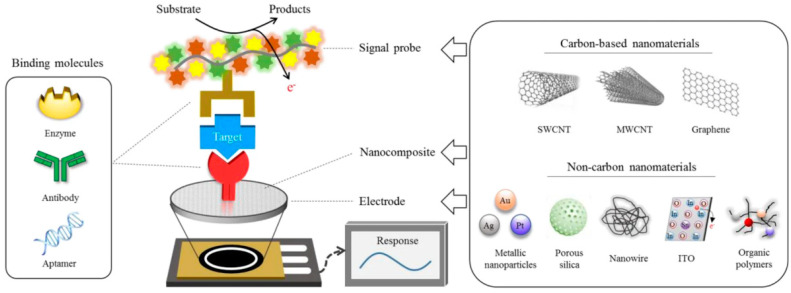
Schematic diagram of the analytical principle of an electrochemical biosensor. Reprinted with permission from Ref. [51]. Copyright 2020, Springer Nature.

Biosensors based on electrical signals change the electrochemical properties of the sensor surface by binding to essential substances in the organism, such as proteins, amino acids, nucleic acids, antibodies, etc. (Figure 5). The development of MXenes for electrochemical biosensors has been intensively investigated because of their excellent properties, such as high conductivity, electrochemical activity, and large surface area. The classification of electrochemical biosensors is as follows: enzyme electrochemical, nucleic acid electrochemical, and immunoelectrochemical biosensing.

#### 3.1.1. Enzyme-Based Electrochemical Biosensing

Enzyme electrochemical biosensors with higher efficiencies and substrate specificities in mild conditions have been extensively explored over the last few years (Table 1). The basic principle is the direct electron transfer (DET) process between the enzyme and the electrode. The immobilisation of enzymes on the bare electrode surface can render the enzymes biologically inactive, making it extremely difficult to perform DET on the electrode’s surface [52]. MXenes can be used as a strategy for enhancing DET because of their large specific surface area, excellent electrical conductivity, and good biocompatibility.

Much of the literature has shown that MXenes or MXenes composite materials can maintain the activity of enzymes after complexing enzymes due to the various properties and unique structures of MXenes. This demonstrates that MXenes can be magnificent structures for enzyme-based biosensors. Xu et al. mixed Ti_3_C_2_ MXene and HRP enzyme directly to fabricate a biosensor for the detection of H_2_O_2_ to analyse the levels of serum samples from AMI patients before and after surgery [53]. Ma et al. fabricated a low detection limit enzyme biosensor for the detection of H_2_O_2_ using a chitosan complex of Ti_3_C_2_ MXene-loaded HRP enzyme and successfully used it to detect trace amounts of H_2_O_2_ in foods [54].

**Table 1:** Enzyme-based electrochemical biosensors for identifying units, target, and analytical parameters.

MXenes Composite	Identify Units	Target	LOD	Range	Ref.
Au/Ti_3_C_2_	glucose oxidase	glucose	5.9 μM	0.1–18 mM	[55]
PLL/Ti_3_C_2_	glucose oxidase	glucose	2.6 μM	4.0–20 µM	[56]
PEDOT: SCX/Ti_3_C_2_T_x_	glucose oxidase	glucose	22.5 μM	0.5–8 mM	[57]
Ti_3_C_2_/Nafions	Horse Radish Peroxidase	H_2_O_2_	1 μM	5–8000 μM	[53]
MXene/chitosan	Horse Radish Peroxidase	H_2_O_2_	0.74 μM	5–1650 μM	[54]
Chit/ChOx/Ti_3_C_2_T_x_	cholesterol oxidase	cholesterol	0.11 nM	0.3–4.5 nM	[58]
Ti_3_C_2_	tyrosinase	phenol	12 nmol L^−1^	50 nM–15.5 μM	[59]
CS-Ti_3_C_2_T_x_	acetylcholinesterase	acetylthiocholine chloride	3 fM	10 nM–10 fM	[60]
GA/Nb_2_CT_x_	acetylcholinesterase	phosmet	144 pM	200 pM–1 μM	[26]

**Figure 6:**
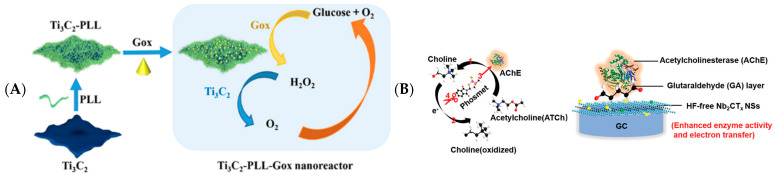
(**A**) Schematic diagram of the detection of the Ti_3_C_2_-PLL-Gox nanoreactor Reprinted with permission from Ref. [56]. Copyright 2020, Elsevier. (**B**) Schematic illustration of the enzymatic inhibition of sulfoxide detection by the HF-free Nb_2_CT_x_/AChE biosensor Reprinted with permission from Ref. [26]. Copyright 2020, Wiley-VCH.

In addition, there are several reports on using MXenes in different compound types of enzymes, such as glucose oxidase [57], cholesterol oxidase [58], acetylcholinesterase [60], tyrosinase [59], etc. Wu et al. proposed a hybrid PLL/Ti_3_C_2_/glucose oxidase glucose biosensor that accelerates the breakdown of H_2_O_2_ generated during glucose oxidation by catalysing a cascade reaction [56] (Figure 6A). Xia et al. developed a Chit/cholesterol oxidase/Ti_3_C_2_T_x_ composite cholesterol oxidase biosensor [55]. Chit/Ti_3_C_2_T_x_ served as a support matrix for immobilising the enzyme. Gold nanoparticles anchored on Ti_3_C_2_T_x_ MXene nanosheets enhanced the electron transfer between the enzyme and the electrode. The relative current sensitivity and LOD were 0.3–4.5 nM and 0.11 nM, respectively. Song et al. derived electrochemical etching to derive fluorine-free Nb_2_CT_x_ with low cytotoxicity and constructed a Nb_2_CT_x_/acetylcholinesterase biosensor to detect sulfoxide [26] (Figure 6B). Moreover, the sensor’s enzymatic activity and electron transfer are superior to the corresponding V_2_C and Ti_3_C_2_ MXenes biosensors. Wu et al. used Ti_3_C_2_ MXene as a new substrate to immobilise tyrosinase and facilitated the direct electron transfer process for the sensitive and rapid detection of phenol [59]. Therefore, Ti_3_C_2_ MXene can be a phenolic biosensor with high recovery and long-term stability. The biosensor exhibits good analytical performance over a wide linear range of 0.05–15.5 μM, with detection limits as low as 12 nM.

The above examples and the contents show that it is feasible to combine enzymes directly on MXenes or with other materials to improve the performance of enzyme electrochemical biosensors.

#### 3.1.2. Nucleic Acid-Based Electrochemical Biosensing

Using nucleic acids as recognition elements allows the specific recognition of the target and the generation of some signal changes [61]. Nucleic acid is a stable and easy-to-handle biomolecule, so it has excellent detection performances [62]. Nucleic-acid-based electrochemical biosensors offer advantages of both nucleic acid probes and electrochemical detection, enabling the sensitive detection of analytes such as nucleic acid, ref. [63] proteins [64], biological molecules [65], inorganic ions [66], and cells [67] (Table 2). Nucleic acid electrochemical biosensors are based on five conformations: double-stranded, triple-stranded, quadruple-stranded, DNA nanostructures, and single-stranded DNA functionalisation (hairpin structure, aptamers, and DNAzyme) [68]. Unlike enzymes, nucleic acids possess little redox capacity. The development of nucleic acid electrochemical biosensors generally relies on molecules with redox properties, such as methylene blue (MB) and ferrocene (Fc), or through charge changes that occur during nucleic acid hybridisation [69]. The nucleic acid electrochemical biosensor has various applications in genetics, clinical medicine, and biosensing due to its rapid detection, simple experimental procedures, high sensitivity, and low cost [70]. There are two types of nucleic acid biosensors.

**Figure 7:**
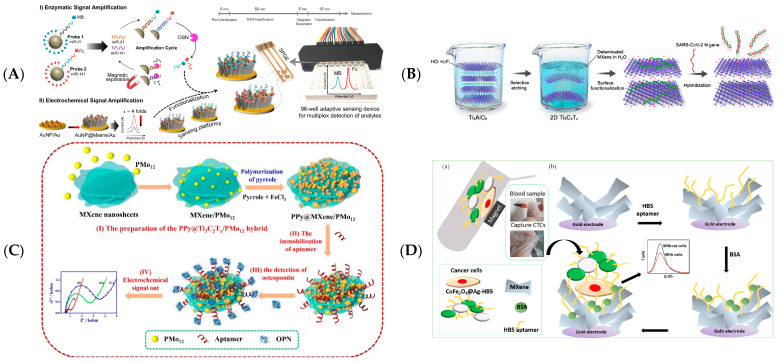
(**A**) Schematic diagram representing the entire assay procedure for multiplex and concurrent detections of miRNA. Reprinted with permission from Ref. [73]. Copyright 2020, Elsevier. (**B**) Schematic of the ssDNA/Ti_3_C_2_T_x_ for the detection of the SARS-Cov-2 nucleocapsid gene. Reprinted with permission from Ref. [74]. Copyright 2022, American Chemical Society. (**C**) Schematic diagram of PPy@Ti_3_C_2_/PMo_12_ aptamer biosensor for OPN detection. Reprinted with permission from Ref. [64]. Copyright 2019, Elsevier. (**D**) Schematic diagram of the MXenes based cell sensor for the detection of SK-BR-3 cells: magnetic cell separation using CoFe_2_O_4_@Ag-HB5 (**a**) and electrochemical cell detection on a functionalised MXenes surface (**b**). Adapted with permission from Ref. [77]. Copyright 2021, Elsevier.

The first type of nucleic acid electrochemical biosensor follows the Watson–Crick pairing principle, which hybridizes a nucleic acid sequence with a complementary nucleic acid sequence through base pairing [61]. The detection principle works by immobilising nucleic acids on the electrode’s surface to capture complementary nucleic acid sequences, thus obtaining an altered electrical signal for specific detection [71]. There are many reports using specific nucleic acid sequences to create biosensors for the detection of disease-predicting miRNAs and DNA, and some electrochemical biosensors have been validated for point-of-care detection. Duan et al. developed a Ti_3_C_2_/FePc QDs MXene nanocomposite nucleic acid biosensor with good biocompatibility [72]. The Ti_3_C_2_/FePc QDs composite material was used as a carrier to detect miRNA-155 by using a change in electrochemical impedance caused by DNA modifications. Mohammadniaei et al. used double screen-printed gold electrodes modified with MXenes and AuNPs and single-stranded DNA-functionalised magnetic particles to detect miRNA-21 and miRNA-141 by using duplex-specific nuclease (DSN) amplification assay strategy [73] (Figure 7A). This biosensor can continue to be upgraded to quantify more analytes, forming a device for point-of-care testing (POC) cancer screening. Chen et al. fabricated a DNA electrochemical biosensor using MXene-based [74]. The surface groups were covered using ssDNA adsorbed on Ti_3_C_2_ MXene to attenuate conductivity. When target DNA and ssDNA are hybridized and desorbed from Ti_3_C_2_ MXene, the fast, simple, and sensitive detection of N-gene sequences in SARS-Cov-2 was possible (Figure 7B). The feasibility of DNA-functionalised MXenes in developing real-time monitoring diagnostic devices for clinical testing can be demonstrated.

**Table 2:** Nucleic acid-based electrochemical biosensors identify units, target, and analytical parameters.

MXenes Composite	Identify Units	Target	LOD	Range	Ref.
MoS_2_ /Au NPs/Ti_3_C_2_	DNA probe	miRNA-182	0.43 fM	1 fM–0.1 nM	[63]
Au/Ti_3_C_2_	DNA probe	miRNA-21, 141	204 aM 138 aM	500 aM–50 nM	[73]
Ti_3_C_2_T_x_ @FePcQDs	DNA probe	miRNA-155	4.3 aM	0.01 fM–10 pM	[72]
MCH/CP/AuNPs/Ti_3_C_2_T_x_	DNA probe	BCR/ABL fusion gene	0.05 fM	0.2 fM–20 nM	[75]
Ti_3_C_2_T_x_	DNA probe	SARS-Cov-2 N gene	10^5^ copies mL^−1^	10^5^–10^9^ copies mL^−1^	[74]
PMo_12_/PPy@Ti_3_C_2_T_x_	Aptamer	Osteopontin	0.98 fg mL^−1^	0.05–10,000 pg mL^−1^	[64]
AuNPs/Ti_3_C_2_	Aptamer	Mucin 1	0.72 pg mL^−1^	5 pg mL^−1^–50 ng mL^−1^	[76]
Ti_3_C_2_	Aptamer	gliotoxin	5 pM	5 pM–10 nM	[65]
Ti_3_C_2_	Aptamer	HER2-positive CTCs	47 cell mL^−1^	20–200 cells mL^−1^	[77]
CoCu-ZIF@ Ti_3_C_2_ CDs	Aptamer	B16-F10 cell	33 cells∙mL^−1^	1 × 10^2^–1× 10^5^ cells∙mL^−1^	[67]
Au@Nb_4_C_3_T_x_	Aptamer	Pb^2+^	4 nM	10 nM–5 μM	[66]

The second nucleic acid electrochemical biosensor uses single-stranded DNA (ssDNA) or RNA to bind to various biomolecules for analyte detection, including proteins, small biomolecules, cells, etc. [78,79]. Electrochemical biosensors made up of aptamers are easy, reliable, quick in responding, low in price, and possess acceptable repeatability [80]. Geng Xue of our research group cleverly used conformational changes of aptamers before and after capturing serotonin to construct an aptamer biosensor [81]. The interaction between aptamer and serotonin was destroyed by guanidine hydrochloride, and 98.2% of the signal was recovered, showing acceptable repeatability. Zhou et al. synthesized intercalating polypyrrole (PPy) Ti_3_C_2_T_x_ MXene and phosphomolybdic acid (PMo_12_) composites with a strong synergistic effect, promoting the anchoring of RNA aptamers on the composites [64] (Figure 7C). The G-quadruplex formed by osteopontin (OPN) and aptamer exhibits stable and high sensitivity, which proves the excellent performance of this MXene composite aptamer biosensor. Li et al. created a nuclease-driven DNA walker cascade signal amplification strategy to construct electrochemical aptamer biosensors on Au nanoparticles/MXene-modified electrodes for mucin 1 [76]. A DNA nanostructure-modified Ti_3_C_2_ MXene nanosheet biosensor was developed by Wang et al. for the detection of gliotoxins [65]. Tetrahedral DNA nanostructures were quickly immobilised on the surface of MXenes nanosheets, thus avoiding the tedious and expensive modification of DNA probes. HB5 aptamer immobilised on the MXenes layer via electrostatic interactions was highly selective for HER-2-positive cells, as reported by Vajhadin et al. Sandwich-like structures formed between magnetically captured cells, and functionalised MXenes electrodes effectively shield electron transfers, allowing quantitative cell detection with changes in the current [77] (Figure 7D).

#### 3.1.3. Immunoelectrochemical Biosensing

Electrochemical immunosensors are coupled to the sensor via antigen–antibody interactions. The accessibility of antibody to a wide range of molecules and the high selectivity and sensitivity renders immunochemical methods valuable for clinical diagnosis. These electrochemical biosensors for bioanalysis have advantages of small reagent volumes, high sensitivity and specificity, and portability [82]. As observed from the contents, immunoelectrochemical biosensors offer tremendous advantages in the specific detection of biomolecules (Table 3).

**Figure 8:**
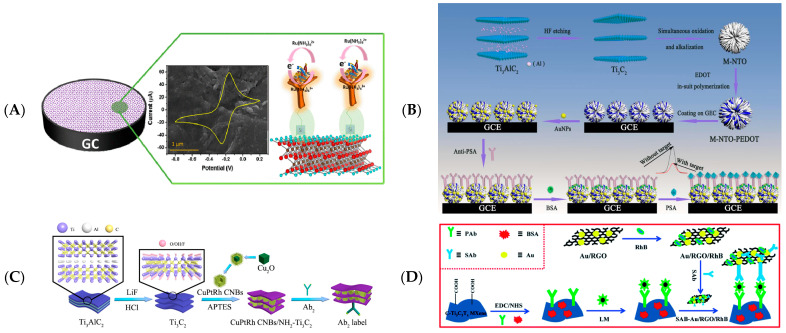
(**A**) Schematic diagram of the detection mechanism of electrochemical CEA. Reprinted with permission from Ref. [84]. Copyright 2018, Elsevier. (**B**) The fabrication and detection steps of the immunosensor. Reprinted with permission from Ref. [86]. Copyright 2020, Elsevier. (**C**) Schematic diagram of the fabrication of the working electrode of the immunosensor. Reprinted with permission from Ref. [87]. Copyright 2020, American Chemical Society. (**D**) Illustrations for constructing the sandwich-like immunosensor of LM based on Ti_3_C_2_T*_x_* MXene. Reprinted with permission from Ref. [88]. Copyright 2021, Royal Society of Chemistry.

In 2018, Kumar et al. fabricated the first MXene-based immunoelectrochemical sensor to detect carcinoembryonic antigens (CEAs) [84]. Aminosilane-functionalised MXenes offered more binding sites for bioreceptors than GCE, and the CEA antigen is better immobilised on Ti_3_C_2_ MXene (Figure 8A). Xu et al. synthesized a composite of 3D sodium titanate nanoribbons, anchored poly(3,4-ethylene dioxythiophene), and gold nanoparticles by oxidizing and alkalizing Ti_3_C_2_ Mxene [86]. The composites described above were used to immobilise prostate-specific substance (PSA) antibodies to create a facile electrochemical label-free immunosensor for the sensitive detection of PSA (Figure 8B).

Dong et al. used a CuPtRh/NH_2_-Ti_3_C_2_ nanocomposite composed of trimetallic hollow CuPtRh cubic nanoboxes (CNBs) and laminated ammoniated Ti_3_C_2_ flakes to fabricate a sandwich-type immunosensor to detect cardiac troponin I (CTnI) [87]. Aminated Ti_3_C_2_ provides abundant binding sites for both CuPtRh CNBs and antibodies, while CuPtRh CNBs can prevent Ti_3_C_2_ from stacking again (Figure 8C). In addition, MXenes can serve to detect bacteria. Niu et al. constructed a sensing platform with carboxylated Ti_3_C_2_T_x_ MXene and rhodamine B/gold/reduced graphene oxide as the signal [88] (Figure 8D). A sandwich electrochemical immunosensing platform for detecting Listeria monocytogenes was also developed by them.

### 3.2. Optical Biosensing

Optical properties, including absorption, transmission, photoluminescence, scattering, and emission, are essential for applying MXenes. The surface groups, doping, and defects affect the energy band’s structure [89]. A thin layer of Ti_3_C_2_T_x_ has been reported to absorb photons in the UV-visible region between 300 and 500 nm with a transmission of 91%. O-functionalised Ti_3_C_2_ MXene has a higher light absorption efficiency [90]. The optical properties are also affected by the thickness of the film and the distance between MXenes layers. Intercalation with hydrazine, urea, methyl ammonium hydroxide, and DMSO changes the interlayer distance of Ti_3_C_2_T_x_, decreasing light transmittance [50].

MXenes have excellent hydrophilicity, biocompatibility, and optical characteristics, making them appropriate for all sorts of biosensing applications. It was discovered as a fluorescence quenching agent and a carrier for biomedical and imaging applications, contributing to high-performance optical biosensors. The interaction of light and materials is central to the optical inspection principle. It identifies samples by non-destructively monitoring changes in the intensity or spectral shift of light [91]. This section will summarize MXenes biosensing applications in photoluminescence, electrochemiluminescence, and photoelectrochemical applications.

#### 3.2.1. Photoluminescence (PL)

MXenes possess features that make MXenes excellent for fluorescence biosensors, such as larger absorption bands, higher energy levels, etc., which causes fluorescence quenches in fluorescent substances [91]. Hence, changes in fluorescence intensity can be employed as indications for biological analytes detection (Table 4). MXene quantum dots (MQDs) are luminous, extremely water-soluble, dispersible, and biocompatible [92]. As a consequence, searching for photoluminescent biosensors based on MXenes and MQDs has emerged as a widespread research issue [93,94].

Because of MXenes’ strong and broad absorption in the visible and near-infrared regions, MXenes generally act as an acceptor designed to quench the fluorescence signal emitted by sensing probes, such as metal nanoclusters, quantum dots, fluorescent dyes, etc. [94]. Due to the two-dimensional planer structure and hydrophilic surface groups of MXenes, the abundant binding sites and hydrophilic groups on MXenes provide more possibilities for biomolecular interactions [44].

**Figure 9:**
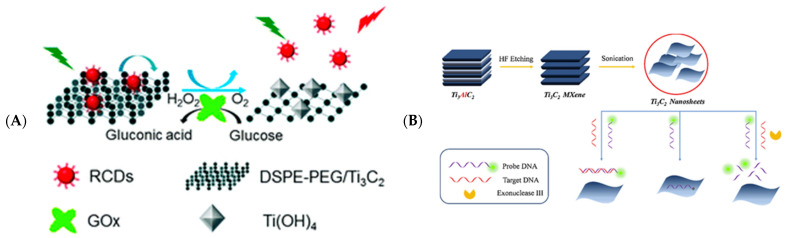
(**A**) Schematic diagram of MXene-based glucose oxidase fluorescent biosensor. Reprinted with permission from Ref. [94]. Copyright 2019, Royal Society of Chemistry. (**B**) Illustration of analysis of HPV-18 type DNA using Ti_3_C_2_ MXene. Reprinted with permission from Ref. [95]. Copyright 2019, Elsevier.

Shi et al. detected glutathione by combining copper nanoclusters (Cu NCs)-functionalised MXenes [99]. MXenes quenches the fluorescence of Cu NCs through the internal filtering effect (IFE), and glutathione can analyze MXenes and Cu NCs, resulting in fluorescence recovery. Ti_3_C_2_ MXene nanosheets combined with red-emitting carbon dots (RCDs) area unit effective and selective fluorescence stimulant sensors were used for glucose detection by Zhu et al. Ti_3_C_2_ nanosheets impassively quenched the fluorescence intensity of RCDs (>96%) through IFE [94] (Figure 9A). Kalkal et al. constructed a fluorescent biosensing system based on Ag/Ti_3_C_2_ to quench the fluorescence signal on antibody/amino-graphene quantum dots [97]. The fluorescence recovered when antigen was added. It can be used to detect neuron-specific enolase with good reproducibility.

MXenes are used as fluorescence quenchers to construct optical sensors for monitoring enzyme activity and biomolecules. Similarly to the previous section, the Fc and MB of the nucleic acid biosensor can be replaced with some fluorescent materials that can be used, which are more practical for this type of biosensor. Zhu et al. reported a Ti_3_C_2_ MXene-based fluorescent biosensor to detect phospholipase D by FRET quenching of rhodamine B (RhB)-labeled phospholipids [98]. Phospholipase D cleaves phospholipids, causing RhB-labeled phospholipids to detach from Ti_3_C_2_ MXenes and re-reflorescence. Peng et al. used the affinity difference between single-stranded and double-stranded DNA on MXenes to construct fluorescent signal detection for human papillomavirus HPV-18 DNA on ultra-thin Ti_3_C_2_ MXene [95] (Figure 9B). Wang et al. presented dual-signal-labelled DNA-functionalised Ti_3_C_2_ MXene nanoprobes to achieve a dual analysis of MUC 1 and miRNA-21 at low concentrations in vitro, and the in situ imaging of MCF-7 breast cancer cells [96]. Furthermore, cell imaging can provide multiple layers of information, such as biomarkers’ expression levels and spatial distribution.

When the thickness dimensions of 2D nanomaterials are less than 100 nm, MXenes can be converted into quantum dots with quantum confinement and optical properties [105]. MQDs, with an average lateral size ranging from 1.8 to 16 nm, can be obtained by hydrothermal processes [100], acidic oxidation, and chemical stripping [106]. Charge transfer is enhanced, and fluorescence is enhanced by utilizing heteroatom doping [101]. MQDs have similar properties to MXenes, such as high dispersion and good biocompatibility. The small band gaps of MXenes can expand their band gap through quantum effects, contributing to their strong fluorescence effect [107]. Some researchers synthesized MQDs that exhibited different fluorescence effects in different solvents under 365 nm UV light irradiation [93,104].

**Figure 10:**
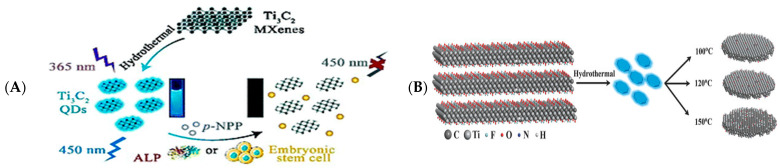
(**A**) Schematic diagram of fluorescence assay of alkaline phosphatase activity of MQDs. Reprinted with permission from Ref. [102]. Copyright 2018, Royal Society of Chemistry. (**B**) Schematic diagram of hydrothermal preparation of MQDs. Reprinted with permission from Ref. [100]. Copyright 2017, Wiley-VCH.

On account of their tunable size, photoluminescence, and photostability, MQDs can be applied as fluorescent probes and can also be functionalised with natural biomolecules [107]. The performance of MQDs as fluorescent agents or signals can be improved, and the application of MXenes in biosensing can be widely expanded [91,108]. MQDs have contributed enormously to detecting metal ions, biomolecules, and cellular imaging. The first MQD-based fluorescence sensor is based on the coordination of Zn^2+^ through hydroxyl groups on the surface of MQDs with selective quenching [100] (Figure 10B). Heteroatom-doped MQDs can be the detector for the fluorescence detection of different metal particles, such as Cu^2+^ [101], Ag^+^, and Mn^2+^ [109].

MQDs can be implemented to detect some biomolecules because they have absorption bands that overlap with the excitation and/or emission spectra of MQDs. Guo et al. designed an MQD-based fluorometric strategy for alkaline phosphatase activities and embryonic stem cell identification [102] (Figure 10A). The effective quenching of MQD fluorescence was obtained by p-nitrophenol produced by the alkaline phosphatase-catalysed dephosphorylation of p-nitrophenyl phosphate. It can also be used as an IFE-based method to analyse ESC biomarker ALP in ESC lysates accurately. Liu et al. described a fluorescent platform for detecting cytochrome c and trypsin [103]. The fluorescence of MQDs was burst by cytochrome c through the IFE. Meanwhile, cytochrome could be degraded by trypsin, and MQDs’ fluorescence could be restored. Chen et al. constructed a fluorescent sensor with the pH-dependent emission of blue fluorescence from MQDs for ratiometric MQDs probes to detect cellular pH [104].

#### 3.2.2. Electrochemiluminescence (ECL)

As a mixture of electrochemistry and optics, electrochemiluminescence is a new method for evaluations and detections. Because of its low background signal, excellent sensitivity, controllability, speed, and low cost, it is frequently employed in biochemistry for proteins, nucleic acids, enzymes, and clinical diagnostics [91]. MXenes have been proven viable as working electrodes for ECL biosensors, with improved ECL characteristics compared to glassy carbon electrodes [110]. The ECL biosensor is well suited for the analysis of nucleic acids or gene fragments, biomolecules, biomarkers, and even cells (Table 5).

**Figure 11:**
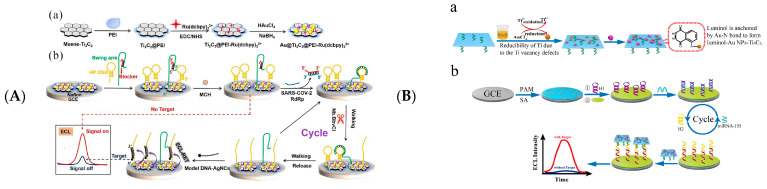

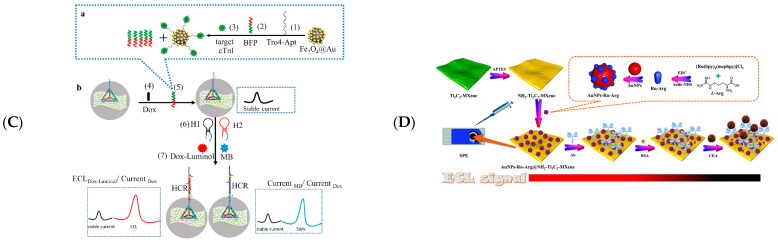
(**A**) Schematic diagram of the preparation of Au@Ti_3_C_2_@PEI-Ru(dcbpy)_3_^2+^ nanocomposites (**a**); Combined unilateral DNA walker amplification strategy based on nanocomposites for ECL biosensor detection of SARS-Cov-2 RdRp gene (**b**). Reprinted with permission from Ref. [113]. Copyright 2021, American Chemistry Society. (**B**) Strategy of stable luminol-Au NPs-Ti_3_C_2_ (**a**) and construction of the proposed ECL biosensor (**b**). Reprinted with permission from Ref. [112]. Copyright 2021, Springer Nature. (**C**) Schematic representation of specific target recognition and BFP release (**a**) and the ratiometric biosensing mechanism of cTnI (**b**). Reprinted with permission from Ref. [118]. Copyright 2021, Elsevier. (**D**) Schematic representation showing the detection principle of the prepared ECL biosensor. Reprinted with permission from Ref. [119]. Copyright 2022, Royal Society of Chemistry.

In 2018, Fang et al. fabricated an ECL biosensor of Ru(bpy)_3_^2+^ functionalised Ti_3_C_2_T_x_ MXene to detect unlabelled single nucleotide mismatches in human urine, using tripropylamine as a co-reactant [111]. Exposed bases in mismatched DNA bind to Ru(bpy)_3_^2+^ on the Ti_3_C_2_T_x_ MXene and are more prone to electrochemical oxidation in enhancing ECL intensities. Zhuang et al. constructed ECL nanoprobes via Ti_3_C_2_T_x_-mediated in situ formations of Au NPs and the anchoring of luminol and utilised the catalytic hairpin assembly (CHA) amplification of signalling to fabricate ECL biosensors for miRNA-155 detection [112] (Figure 11A). Yao et al. detected the SARS-Cov-2 gene by MXenes/PEI adsorbed Au and Ru(bpy)_3_^2+^ DNA walkers [113]. After the DNA walker excised hairpin DNA under the action of Nb.BbvCl endonuclease, template DNA-Ag hybridized with hairpin DNA and decreased the signal of ECL (Figure 11B). Zhang et al. modified DNA probes on MXenes/PEI composites by Ru(bpy)_3_^2+^ and AuNP and used the CRISPR-Cas12a strategy to construct an ECL signal on/off biosensor for the detection of SARS-Cov-2 (RdRp) gene [114].

**Table 5:** MXene-based electrochemiluminescence biosensors identify units, target, and analytical parameters.

MXenes Composite	Identify Units	Target	LOD	Range	Ref.
g-C_3_N_4_/Ti_3_C_2_	Ti_3_C_2_	Protein Kinase	1.0 mU mL^−1^	0.015–40 U mL^−1^	[115]
Ti_3_C_2_T_x_	Ru(bpy)_3_^2+^	nucleotide mismatch	5 nM	-	[111]
Au@Ti_3_C_2_@PEI-Ru(dcbpy)_3_^2+^	Model DNA-AgNCs	SARS-Cov-2 Gene	0.21 fM	1 fM–100 pM	[113]
AuNPs/Ti_3_C_2_/Luminol	sDNA	miRNA-155	0.15 fM	0.3 fM–1 nM	[112]
Ru@Ti_3_C_2_@AuNPs	Fc-DNA	SARS-Cov-2 gene	12.8 aM	-	[114]
Ti_3_C_2_/PEI	aptamer	MCF-7	125 particles μL^−1^	5 × 10^2^–5 × 10^6^ particles μL^−1^	[116]
Ti_3_C_2_/Au	aptamer	CD63	30 particles μL^−1^	10^2^–10^5^ particles μL^−1^	[117]
AuNPs/Ti_3_C_2_	aptamer	cardiac troponin I	0.04 fM	0.1 fM–1 pM	[118]
AuNPs-Ru-Arg@Ti_3_C_2_	antibody	CEA	1.5 pg mL^−1^	0.01–150 ng mL^−1^	[119]
R6G-Ti_3_C_2_T_x_@AuNRs/ABEI	antibody	Vibrio vulnificus	1 CFU mL^−1^	1–10^8^ CFU mL^−1^	[120]

Strategies for detecting biomolecules can be implemented with aptamers, resulting in higher ECL signal intensities. Sun et al. proposed PEI-functionalised MXenes and g-C_3_N_4_ composites as detection probes, and kemptide chelated with Ti in the composites after protein kinase A (PKA) phosphorylation to promote electron transfers at the electrode’s interface, enhancing strategies for ECL signalling [115]. Moreover, this biosensor enables inhibitor screening and PKA activity monitoring in MCF-7 cell lysates. Mi et al. reported a method for the quantitative detection of cardiac troponin (CTnI) by electrochemical and ECL dual signals using tetrahedral DNAs (TDs) and in situ hybrid chain reaction (HPR) on Au/Ti_3_C_2_ MXene [118] (Figure 11C). Both ECL Dox-Luminol/Current Dox and Current MB/Current Dox dual signals can be used for the quantitative detection of CTnI, which is expected to be used in screen critically ill patients with COVID-19. Zhang et al. used MXenes to generate AuNPs in situ and modified aptamers and constructed an ECL biosensor to detect exosomes CD63 [117]. Zhang and colleagues developed an exosome-selective ECL biosensor using aptamer-modified Ti_3_C_2_ MXenes as probes with an LOD of 125 μL particles^−1^ [116].

Immunochemical methods are also highly selective and sensitive in the field of ECL. Luo et al. constructed an MXene-based substrate using [Ru(bpy)_2_(mcpbpy)]Cl_2_ and L-arginine as co-reactants to detect carcinoembryonic proteins (CEA) by antigen [119]. Upon antigen binding to the antibody, spatial site resistance leads to a decline in the rate of electron transfer and electrolyte diffusion at the electrode’s surface, resulting in a decrease in ECL signal intensities (Figure 11D). Wei et al. constructed an ECL/SERS dual-signal biosensor to detect the causative agent of Vibrio vulnificus (VV) [120]. The pathogenic bacteria. VV is captured by Fe_3_O_4_@Ab_1_ as the capture unit. Ab_2_, R6G, and ABEI bind to AuNR as the signal unit to capture VV through Au-S and Au-N, forming a Faraday cage structure.

#### 3.2.3. Photoelectrochemical (PEC)

Similarly to electrochemiluminescence sensing, photoelectrochemical sensing is a practical analytical method that integrates optical and electrochemical analyses. MXenes also promise photoelectrochemical sensors with their excellent optical and electronic properties [121] (Table 6).

Li et al. took advantage of Ti_3_C_2_ MXene, readily forming PN junctions with photosensitive semiconductors and, therefore, used Ti_3_C_2_/Cu_2_O heterostructures for the high-sensitivity detection of glucose [122]. The in situ growth of Cu_2_O on MXenes improves photoelectrochemical performances compared to pure Cu_2_O. In order to improve the photocurrent conversion efficiency and detection sensitivity of glucose, a Z-type heterostructure based on TiO_2_/Ti_3_C_2_T_x_/Cu_2_O was proposed in addition to the construction of a Schottky-junction-based PEC sensor [123]. A DNA probe can also achieve the label-free PEC determination of methyltransferase (MTase) for label-free Bi_2_S_3_/Ti_3_C_2_ PN junctions [124]. For exosomes, the enzyme-induced deposition of CdS on Ti_3_C_2_ MXene forms a Ti_3_C_2_ MXene/CdS composite, creating a built-in electric field in the tight interface between CdS and Ti_3_C_2_ MXene, enabling highly accurate detection [125]. In addition to this, the use of Ti_3_C_2_@ReS_2_ to immobilise DNA probes and perform specific PEC detections of miRNA-141 has excellent performance [126]. For the detection of 5hmCTP on APTES/Ti_3_C_2_, the use of antibodies for PEC is also feasible [127]. Chen et al. developed a photoelectrochemical biosensor for the sensitive and selective detection of glutathione based on MQDs [128].

### 3.3. Other Biosensing

#### 3.3.1. Wearable Biosensing

The covalent between the M transition metal and the X element in MXenes, the terminal surface groups, and the thickness of the atomic layers resulted in excellent mechanical properties [3,18]. Numerous theoretical findings on the mechanical properties of MXenes have been reported. Kurtoglu et al. predicted a higher elasticity coefficients for various pristine and functionalised MXenes than their precursors due to the greater density of charge density in the M_n+1_X_n_ layer [129]. The excellent mechanical properties provide favourable conditions for fabricating wearable biosensors.

Some studies have used wearable nanoelectronics to detect health-related physiological activities, such as physical or chemical stimulation, micropressure, and changes in physiological signals. Stretchable mechanical properties, high gauge factor, flexible materials integrating flexible bio-electronic interfaces, and miniaturized signalling systems need to be investigated to meet the required sensitivity of sensor devices and to improve the usability of wearable devices [130]. Recently, ultrathin MXenes comprised high-performance materials for stretchable and bendable conductive coatings [131] (Table 7). Conductive and conformal MXenes multilayers can withstand up to 2.5 mm bending and 40% tensile stretching, with recoverable electrical resistances, while maintaining a conductivity of 2000 S m^−1^ [10].

**Figure 12:**
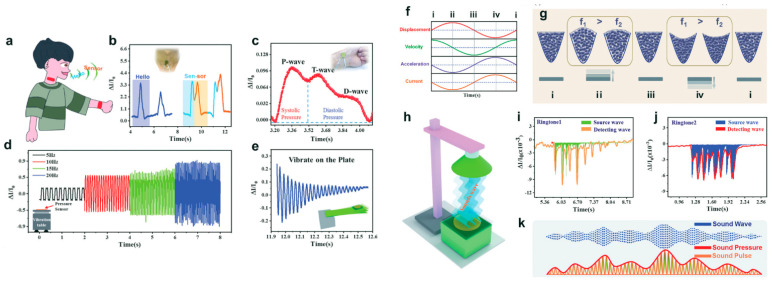
Schematic diagram of a pressure sensor as a wearable device for physiological signal detection (**a**). Corresponding signals for saying “hello” and “sensor” (**b**). Corresponding signal for the pulse on the medial wrist (**c**). Corresponding signal of the pressure sensor vibrating at different frequencies on the shaker (**d**). Corresponding signals of the pressure sensor vibrating on plate (**e**). Corresponding relationship between current values, displacement, velocity and acceleration in the vibration mode (**f**). Schematic diagram of the density of MXenes at different vibration stages (**g**). Diagram of a sound wave hitting a pressure transducer (**h**). Schematic diagram of the acoustic pressure on the sensor surface associated with sound waves and sound pulses (**i**). Corresponding detection signals and source waves for the two ringtones (**j**–**k**). Reprinted with permission from Ref. [140]. Copyright 2020, Wiley-VCH.

Piezoresistive wearable biosensors are designed to detect weak movements of the human body using stretch changes in materials. The development of MXene-based piezoresistive biosensors has been reported to change the resistance of the biosensor by varying the MXene’s layer spacing under external pressure [138]. The biosensor can monitor physical stimulation processes, such as blinking, throat swallowing, and knee bending release through electrical current. Strain sensors were also fabricated by using Ti_3_C_2_ MXenes nanocomposites with single-walled carbon nanotubes (SWCNT) obtained by layer-by-layer (LBL) spraying [139]. The multifunctional force-sensing sensor for acoustic monitoring consists of two Au electrodes on a polyethylene terephthalate (PET) substrate at the top and bottom, an intermediate MXenes layer, and a fingerprint structure on the substrate in a combined arrangement [140]. The manufactured sensor is versatile and capable of sensing sound, micro-motion, and acceleration in a single device. This biosensor can be flexibly attached to a person’s throat and wrist and is used to detect a person’s vocalisation and pulse. The sensor can record relevant peaks when saying “hello” and “sensor” or when detecting a steady heartbeat pulse signal. The biosensor has shown excellent sensitivity in detecting subtle human activity and other weak stresses (Figure 12). It offers a new research direction for portable and wearable sensing devices in biosensing and human behaviour analyses.

**Figure 13:**
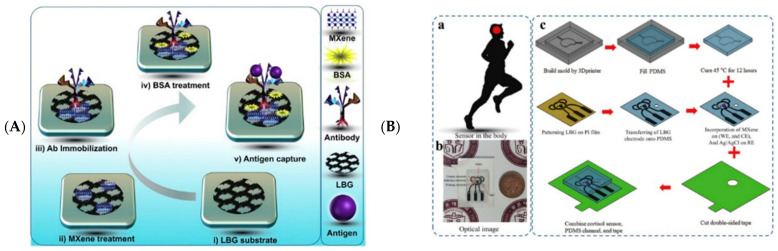
(**A**) Schematic representation of the Ti_3_C_2_Tx MXene-loaded/LBG-based cortisol biomarker assay. (**B**) Patch sensor attached to the body position (**a**), optical image of the wearable patch (**b**) and fabrication sequence of a wearable patch cortisol sensor integrated with a microfluidic system (**c**). Reprinted with permission from Ref. [132]. Copyright 2020, Elsevier.

Wearable microfluidic biosensors were originally designed to integrate biological identifiers (enzymes, nucleic acids, enzymes, or cellular receptors) into the sensor operation. Non-invasive biomarker detection platforms via biofluids such as sweat, saliva, tears, or interstitial fluid are more practical [141]. Such wearable sensors provide real-time biochemical information about the wearer’s health and offer effective disease detection and body function management [142]. A 3D electrode network electrochemical impedance immunosensor based on loaded laser-burned graphene (LBG) loaded with Ti_3_C_2_T_x_ was fabricated for the non-invasive monitoring of cortisol biomarkers in human sweat [132]. The sensor has a detection limit and linearity of 88 pM and 0.01–100 nM, respectively (Figure 13). In addition, microfluidic wearable biosensors can be used to detect K^+^, Na^+^ ions [135,136], glucose, lactate [134,143], pH, and other human biochemical information in biological fluids [137].

In various wearable biosensors, sensing electrodes play an essential role in the design of wearable biosensors. MXenes offer the ability to immobilise biomolecules as a sensitive detection platform. Nevertheless, the mechanical friction and deformation of wearable devices against human skin over time leads to mechanical failure and requires a re-structuring of the device. Moreover, the attainment of signal-to-noise ratios and the stability required to achieve this device are highly challenging [133].

#### 3.3.2. Surface-Enhanced Raman Spectroscopy (SERS)

The hydrophilic nature of the MXenes surface provides a good site for Raman labelling. It serves as a potential material for SERS and provides an effective method for the ultra-sensitive determination of targets (Table 8). Sarycheva et al. showed that composites of metals and MXenes could be used as SERS subestrates for rhodamine 6G [144]. Integrating noble metal nanoparticles with MXenes exhibits an empathetic, sensitive SERS response in detecting several common dye molecules. This extends MXenes composites for visible light SERS in the sensor field. A reliable substrate for MXenes/AuNR composites was prepared by Xie et al. with high sensitivities for determining common organic dyes, such as Rh6G, crystalline violet, and peacock green [145]. It can detect organic contaminants and shows high sensitivities for more complex organic pesticides and contaminants. A ratiometric SERS aptamer sensor for ochratoxin A was developed by Zhao et al. 2-Mercaptobenzimidazole-5-carboxylic acid ligands and Au-Ag Janus nanoparticles were used as Raman signal molecules to amplify the SERS signal efficiently [146]. Liu et al. used the Ti_3_C_2_T_x_-PDDA-Ag NPs hybrid platform as a sensitive and homogeneous biosensor for the label-free quantification of the biomolecule adenine based on the SERS method [147]. These studies demonstrate that MXenes can be well-suited for SERS.

#### 3.3.3. Surface Plasmon Resonance (SPR)

SPR sensing is a non-destructive, label-free, real-time detection method. Nanomaterials modify the sensor’s surface to enhance the signal and demonstrate high sensitivities to low concentration targets [148] (Table 9). MXenes-based SPR biosensors for the ultra-sensitive detection of carcinoembryonic antigens (CEAs) were fabricated by Wu et al. These SPR biosensors demonstrate good reproducibility and high selectivity in human serum samples, providing a potential method for early clinical diagnoses and cancer surveillance [149]. Their group also constructed SPR biosensors for CEA using amino-functionalised MXenes [150]. MXenes were assembled on Au films to immobilise monoclonal anti-CEA antibody sensing materials covalently. MXenes were used as a substrate for binding hollow gold nanoparticles (HGNPs), and they were modified with SPA. MXenes/HGNPs/SPA/Ab_2_ nanocomplexes act as signal enhancers for SPR-sensing components. The sensor offers a wide linear detection range, ultra-low detection limits, and good selectivity for CEA in human serums. Chen et al. anchored targeting aptamers with thiol-modified niobium carbide MXene quantum dots and could specifically bind the SARS-Cov-2 N gene, resulting in a change in the SPR signal for laser irradiation at a wavelength of 633 nm [151]. The above studies indicate the development of MXene-based biomarker sensing chips or devices in the field of SPR to broaden the area of MXenes biosensing applications.

## 4. Conclusions and Outlook

From its discovery to the present moment, MXenes, an emerging two-dimensional biosensing material, has achieved unprecedented rapid development. Although the development time is relatively short, synthesis methods of MXenes are constantly innovating and developing towards the direction of green environmental protection, simplicity and convenience, and controllable surface groups. MXenes are known for their tunable surface groups; good hydrophilicity and biocompatibility; excellent mechanical, electrical, and optical properties; and specific morphological structures. They have become a focus of research in electrochemical and optical biosensing.

HF synthesis is undoubtedly convenient and quick, giving access to abundant hydrophilic surface groups. However, using F^−^ can cause a certain amount of pollution to humans, the environment, and even the ecosystem. For the synthesis of MXenes, synthetic research has always progressed in the direction of less or no fluorine. Different synthetic methods and raw materials can adjust different surface groups. The structure determines the properties, and mechanical, hydrophilic, biocompatible, electrical, and optical properties can be adjusted according to different research needs using different synthetic routes.

From its large specific surface area, high electrical conductivity, large surface groups, and fast electron transfer properties, MXenes are often used as modification and immobilisation materials in electrochemical biosensors to improve electrocatalytic performances and detection sensitivities and to reduce redox potentials in order to obtain high-performance composites. However, most of the current MXene-based electrochemical biosensors use composite materials adsorbed on MXene’s surface. There are relatively few reports on surface groups on MXenes as the adsorption media for the adsorption of biomolecules to prepare electrochemical biosensors. With the development of the tunable functionalisation of MXenes surface groups, we believe there is more significant potential for the direct binding of MXenes surfaces to biomolecules, such as proteins and nucleic acids. Although optical biosensors have been less studied than electrochemical biosensors, optical sensing is also a vital detection strategy in biosensing. By benefitting from the fluorescence quenching effect of MXenes and MQD’s fluorescence enhancement effect, photoluminescence has been reported relatively more often in optical biosensing work. From the above studies, it can be concluded that nucleic acid-based biosensors are less selective than enzyme and immune-based biosensors. However, nucleic acids are smaller than antibodies and enzymes, so nucleic-acid-based biosensors have a higher density of surface modifications and have a higher sensitivity and reproducibility.

In contrast, SPR, SERS, and other optical sensing have been reported less often. Even so, the sparseness and singularity of functional groups on MXene’s surface led to the reduced binding of biomolecules or other organic molecules. Meanwhile, the study of MQDs with high fluorescence efficiency, quantum yield, and different luminescence wavelengths has become an urgent problem.

About the main challenges mentioned above, we believe that several aspects can be studied for future developments and trends in MXenes biosensing.

(1) MXenes and MQDs synthesis methods are supposed to develop with adjustable size and surface groups. It is necessary to study further study the interaction between the composition, structure, properties, and biomolecules of MXenes in order to understand the appropriate MXene-adaptation-related biosensors that can be developed according to the actual situation. Furthermore, it can provide theoretical support for the development and development of MXenes morphological structure, size, and surface groups, and the study of interaction with biomolecules with the help of machine learning methods.
(2) The direct construction of biosensors on MXenes surfaces: The abundant surface groups of MXenes can be complexed or combined with many biomolecules, and enzyme-based electrochemical sensors have been partially verified. Nevertheless, there are relatively few studies on nucleic acid, antibodies, and other biomolecules combined with MXenes and constructed in sensors. Moreover, the natural binding process can shorten the experimental time and reduce errors caused by too many experimental procedures. At the same time, it is also very convenient for developed point-of-care tests. During the COVID-19 epidemic, point-of-care was necessary for rapid diagnoses and the timely treatment of patients.
(3) Utilise the catalytic properties and the reduction of MXenes: For electrochemical biosensing, MXenes can reduce precious metal nanoparticles in situ. They can also be used to catalyse redox reactions of O_2_ in systems that promote free radical reactions in ECL, and improving the ECL signal is well worth exploring. MXenes are also used as semiconductor materials during electrochemical reactions and can be used to enhance the electrochemical signal. Therefore, reducing the detection limit, extending the detection range, and improving the sensitivity during electrochemical biosensing are possible.
(4) Develop MQDs with high fluorescence efficiency, quantum yield, and different fluorescence emission wavelengths: As a result, the sensitivity and detection limit of fluorescence detections can be improved, and they can be used in optical biosensors with different wavelengths. MQDs can be extended to cell imaging, photothermal therapy, and other biomedical tissue applications.

## Figures and Tables

**Table 3:** Immunoelectrochemical biosensors identify units, target, and analytical parameters.

MXenes Composite	Identify Units	Target	LOD	Range	Ref.
Ti_3_C_2_@CuAu-LDH	Antibody	Carcinoembryonic antigen	0.033 pg mL^−1^	0.0001–80 ng mL^−1^	[83]
Ti_3_C_2_	Antibody	CEA	18 fg mL^−1^	0.0001–2000 ngmL^−1^	[84]
Au/Ti_3_C_2_T_x_	Antibody	PSA	3.0 fg mL^−1^	0.01–1.0 pg mL^−1^	[85]
M/NTO/PEDOT/AuNPs	Antibody	PSA	0.03 pg L^−1^	0.0001–20 ng mL^−1^	[86]
CuPtRh/NH_2_-Ti_3_C_2_	Antibody	cardiac troponin I	8.3 fg mL^−1^	25 fg mL^−1^–100 ng mL^−1^	[87]

**Table 4:** MXene-based photoluminescence biosensors identify units, target, and analytical parameters.

MXenes Composite	Identify Units	Target	LOD	Range	Ref.
DSPE-PEG/Ti_3_C_2_	Gox/RCDs	Glucose	50 μM	0.1–20 mM	[94]
Ti_3_C_2_	DNA probe	HPV-18 DNA	100 pM	0.5 nM–50 nM	[95]
Ti_3_C_2_	Aptamer, DNA probe	MUC1, miRNA-21	6 nM, 0.8 nM	0–60 nM, 0–25 nM	[96]
Ag@Ti3C_2_/GQDs	Antibody NSE	neuron-specific enolase (NSE)	0.05 pg mL^−1^	0.0001–1500 ng mL^−1^	[97]
PL-Ti_3_C_2_	rhodamine B	Phospholipase D	0.10 U L^−1^	0.5–50 U L^−1^	[98]
Ti_3_C_2_T_x_	quantum dots	glutathione	3.0 μM	5.0–100 μM	[99]
Ti3C_2_ QD	quantum dots	RAW264.7 cells, Zn^2+^	-	-	[100]
N,P-Ti3C_2_ QDs	quantum dots	Cu^2+^	2 μM	2–100 μM, 250–5000 μM	[101]
Ti_3_C_2_ MQDs	quantum dots	alkaline phosphatase	0.02 U L^−1^	0.1–2.0 U L^−1^	[102]
epsilon-poly-L-lysine (PLL)/Ti3C_2_ QDs	quantum dots	cytochrome c and trypsin	20.5 nM and 0.1 μg mL^−1^	0.2–40 μM and 0.5–80.0 μg mL^−1^	[103]
Ti_3_C_2_ QDs	quantum dots	Intracellular pH	-	pH 6.0–8.0	[104]

**Table 6:** MXene-based photoelectrochemical biosensors identify units, target, and analytical parameters.

MXenes Composite	Identify Units	Target	LOD	Range	Ref.
Ti_3_C_2_/Cu_2_O	non-enzymatic	glucose	0.17 nM	0.5 nM–0.5 mM	[122]
TiO_2_/Ti_3_C_2_T_x_/Cu_2_O	non-enzymatic	glucose	33.75 nM	100 nM–10 μM	[123]
Bi_2_S_3_/Ti_3_C_2_	DNA probe	methyltransferase	0.003 U mL^−1^	0.01–30 U mL^−1^	[124]
Ti_3_C_2_/CdS	Aptamer	Exosomes	7.875 × 10^4^ particles mL^−1^	7.3 × 10^5^–3.285 × 10^8^ particles mL^−1^	[125]
Ti_3_C_2_@ReS_2_	DNA probe	miRNA-141	2.4 aM	0.1 fM–1 nM	[126]
APTES/ Ti_3_C_2_	Antibody	5hmCTP	4.21 pM	0.008–100 nM	[127]
(IOPCs)/Ti_3_C_2_ QDs	quantum dots	glutathione	9.0 nM	0.1–1000 μM	[128]

**Table 7:** MXene-based wearable biosensors identify units, target, and analytical parameters.

MXenes Composite	Detection Technology	Target	LOD	Range	Ref.
Ti_3_C_2_T_x_/LBG/PDMS	Vitro Perspiration Analysis	cortisol	88 pM	0.01–100 nM	[132]
Ti_3_C_2_T_x_/PB	Vitro Perspiration Analysis	glucose and lactate	35.3 and 11.4 µA mm^−1^ cm^−2^	-	[133]
Ti_3_C_2_T_x_/MB	Vitro Perspiration Analysis	glucose and lactate	2.4 nA μM^−1^ and 0.49 μA mM^−1^	-	[134]
Ti_3_C_2_T_x_/MWNTS	Vitro Perspiration Analysis	K^+^	-	1–32 mM	[135]
Ti_3_C_2_T_x_	Vitro Perspiration Analysis	Na^+^, protein	-	-	[136]
F-Ti_3_C_2_T_x_/PANI	Vitro Perspiration Analysis	human sweat pH	-	-	[137]

**Table 8:** MXene-based other biosensors identify units, target, and analytical parameters.

MXenes Composite	Detection Technology	Target	LOD	Range	Ref.
Ti_3_C_2_T_x_/AuNRs	-	R6G, crystal violet, malachite green	1 pM, 1 pM, 10 nM	-	[145]
Ti_3_C_2_O_2_	Aptamer	Ochratoxin A	1.28 pM	-	[146]
Ti_3_C_2_T_x_-PDDA-AgNPs	Electrostatic	Dopamine	10 nM	5 μM–50 nM	[147]
R6G-Ti_3_C_2_T_x_@AuNRs/ABEI	Antibody Vibrio vulnificus	Vibrio vulnificus	10^2^ CFU mL^−1^	10^2^–10^8^ CFU mL^−1^	[120]

**Table 9:** MXene-based SPR biosensors identify units, target, and analytical parameters.

MXenes Composite	Detection Technology	Target	LOD	Range	Ref.
Ti_3_C_2_/AuNPs	Antibody	carcinoembryonic antigen	0.2 fM–20 nM	0.07 fM	[149]
APTES/Ti_3_C_2_/HGNPs	Antibody	carcinoembryonic antigen	0.001–1000 pM	0.15 fM	[150]
Nb_2_C-SH QDs	Aptamer	N-gene of SARS CoV-2	4.9 pg mL^−1^	0.05–100 ng mL^−1^	[151]

## Data Availability

Not applicable.
